# Body Fluid Biomarkers for Alzheimer’s Disease—An Up-To-Date Overview

**DOI:** 10.3390/biomedicines8100421

**Published:** 2020-10-15

**Authors:** Adrian Florian Bălașa, Cristina Chircov, Alexandru Mihai Grumezescu

**Affiliations:** 1 Târgu Mures, Emergency Clinical Hospital, “George Emil Palade” University of Medicine, Pharmacy, Science and Technology of Târgu Mures, RO-540142 Târgu Mures, Romania; adrian.balasa@yahoo.fr; 2 Faculty of Applied Chemistry and Materials Science, University Politehnica of Bucharest, RO-060042 Bucharest, Romania; cristina.chircov@yahoo.com

**Keywords:** neurodegeneration, Alzheimer’s disease, neurofibrillary tangles, diagnosis methods, biomarkers

## Abstract

Neurodegeneration is a highly complex process which is associated with a variety of molecular mechanisms related to ageing. Among neurodegenerative disorders, Alzheimer’s disease (AD) is the most common, affecting more than 45 million individuals. The underlying mechanisms involve amyloid plaques and neurofibrillary tangles (NFTs) deposition, which will subsequently lead to oxidative stress, chronic neuroinflammation, neuron dysfunction, and neurodegeneration. The current diagnosis methods are still limited in regard to the possibility of the accurate and early detection of the diseases. Therefore, research has shifted towards the identification of novel biomarkers and matrices as biomarker sources, beyond amyloid-β and tau protein levels within the cerebrospinal fluid (CSF), that could improve AD diagnosis. In this context, the aim of this paper is to provide an overview of both conventional and novel biomarkers for AD found within body fluids, including CSF, blood, saliva, urine, tears, and olfactory fluids.

## 1. Introduction

Neurodegeneration is a complex process that encompasses several different molecular pathways and a multifaceted interplay between a variety of regulatory factors [1,2]. It is characterized by a progressive and irreversible neuronal loss from the specific brain and spinal cord regions, mainly the nuclei of the base within the subcortical areas and the cerebral cortex, consequently leading to damage and dysfunction manifested through cognitive and motor dysfunctions [2,3,4]. Generally, the causal factors include the following: oxidative stress and free radical formation; protein misfolding, oligomerization and aggregation; mitochondrial dysfunction, axonal transport deficits and abnormal neuron–glial interactions; calcium deregulation, phosphorylation impairment; neuroinflammation; DNA damage and aberrant RNA processing [2,5,6].

Neurodegeneration is the underlying factor for many debilitating and incurable age-dependent disorders [3,7,8]. The prevalence of neurodegenerative disorders is continuously increasing as a consequence of the dramatic rise in life expectancy due to scientific achievements and progress, thus posing a significant threat to human health [5,7,9,10]. Moreover, neurodegeneration is associated with various neurodegenerative, neurotraumatic, and neuropsychiatric disorders, with considerably diverse pathophysiology, including memory and cognitive impairments, muscle weakness and/or paralysis, abnormal control of the voluntary movement, seizures, confusion, and pain [2,4,7,11,12,13]. Specifically, such diseases vary from progressive degenerative disorders, including Alzheimer’s disease (AD), Parkinson’s disease, Huntington’s disease, amyotrophic lateral sclerosis and multiple sclerosis [4,5,10,14,15], to acute traumatic injuries, such as traumatic brain injury, stroke or spinal cord injury [15,16]. 

Among them, AD is the most common neurodegenerative disorder, affecting more than 45 million individuals worldwide and is expected to reach 60 million by 2030 due to the increase in the elderly population [17,18]. AD is characterized by the progressive death of cholinergic neurons within the hippocampal and cortical regions, the consequent atrophy, abnormal neurotransmission and loss of synapses, and neurodegeneration [4,18,19,20,21]. At molecular levels, the underlying mechanisms of AD involve the extracellular deposition of amyloid-β (Aβ) peptides, known as amyloid plaques, and the intracellular formation of hyperphosphorylated tau (Tubulin Associated Unit) protein aggregates, known as neurofibrillary tangles (NFTs), which subsequently induce oxidative stress, chronic neuroinflammation, neuron dysfunction, and neurodegeneration [4,10,17,19,22,23].

In addition to cognitive tests, the current diagnostic methods rely on imaging techniques [24,25,26,27] and cerebrospinal fluid (CSF) assays. On one hand, the purpose of the neuroimaging methods is assessing the hippocampal atrophy through magnetic resonance imaging and the cortical Aβ deposition through positron emission tomography. On the other hand, CSF analyses aim to provide quantitative measurements of Aβ and tau protein levels as AD biomarkers [18]. However, the available methods are expensive, relatively invasive [18,28], and have low sensitivity and specificity, which result in the risks of either overdiagnosis or undiagnosed, misattributed, or dismissed and ignored symptoms [29,30]. Additionally, as there is a serious lack of AD diagnosis assays at all illness stages, patients are generally diagnosed late, which places a great burden on the health systems [17,18]. Therefore, the development of novel methods of AD early detection and accurate diagnosis is essential [17,29].

Detection strategies based on novel biomarkers beyond Aβ and tau protein could represent a promising solution for the early diagnosis of AD [18]. However, as no single biomarker can be used to accurately diagnose AD, a combination of biomarkers could significantly increase diagnostic accuracy [30,31]. Moreover, such biomarkers should ideally be easy to sample and should be measurable through simple and cost-efficient methods and at all stages of the disease, allowing for standardization processes [30,32]. In this context, the aim of this manuscript is to provide an up-to-date overview of both conventional and novel AD biomarkers, which could play fundamental roles in its accurate and timely diagnosis.

## 2. Cerebrospinal Fluid Biomarkers

Biomarkers can be described as molecules that can be detected and quantified within body fluids, such as blood, CSF, urine or saliva, and changes in their levels or activity are generally associated with different pathologies. Offering the possibility of early disease diagnosis, most biomarkers involve the measurement of structural, metabolic or enzymatic proteins and should be non-invasive, easy to use, and cost-efficient [33,34].

Residing in the subarachnoid space and ventricular system of the brain and spinal cord, the CSF is a fundamental neuropathology indicator as it carries the brain’s interstitial fluid across the ventricular ependymal lining, and thus it reflects any biochemical change within the brain [35,36]. Moreover, as the blood–CSF barrier restricts the transport of molecules and proteins, the CSF is isolated from the peripheral system. Thus, it is a useful matrix for the detection of neurodegenerative disorder markers, providing the tools for disease screening, prognosis and monitoring [37,38,39]. Among them, AD biomarkers have received a great deal of clinical interest, allowing for the depiction of AD pathology [35,39,40]. Furthermore, such biomarkers could also be applied for the diagnosis of mild cognitive impairment (MCI), the transitional phase from normal cognition to dementia, that generally manifests as a silent pre-clinical phase in 6–15% of AD patients [40,41,42]. 

The CSF biomarkers most indicative of AD are associated with the main pathological changes in the brain, namely the deposition of extracellular Aβ plaques, the formation of NFTs, and the loss of neurons [37]. Thus, the biomarkers that have received clinical attention for AD diagnosis are Aβ, total-tau (T-tau) and phosphorylated-tau (P-tau), as they are recognized by the International Working Group (IWG) 2 Criteria for AD and the National Institute on Aging-Alzheimer’s Association (NIA-AA) Criteria for AD and MCI associated with AD [35,39].

**Figure 1:**
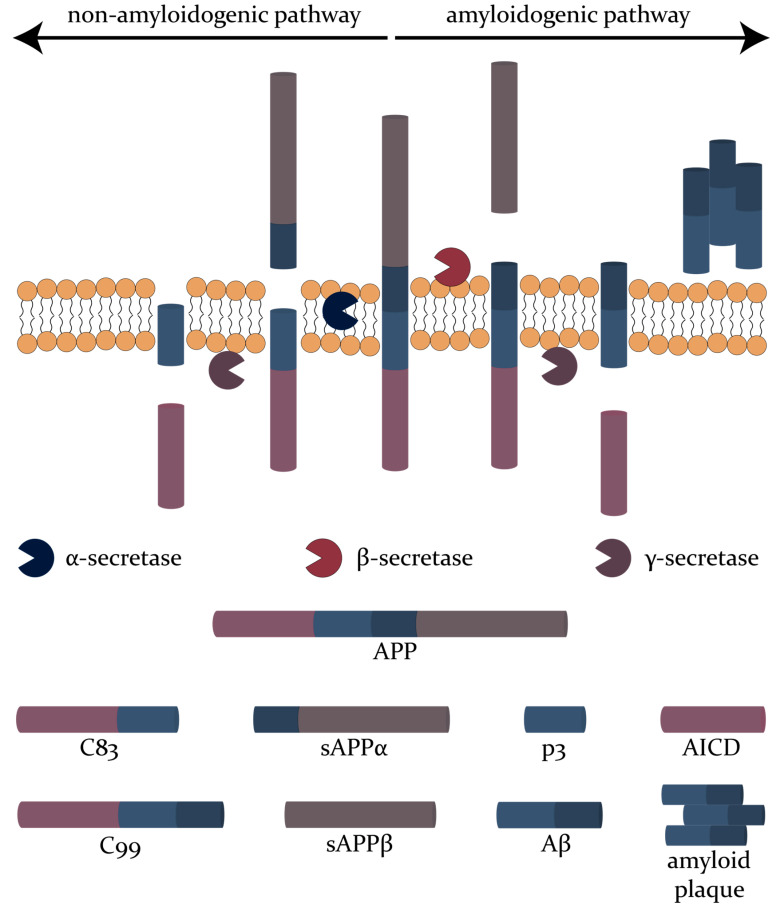
The non-amyloidogenic and amyloidogenic pathways involved in the enzymatic processing of APP.

Generally, the transmembrane protein amyloid precursor protein (APP) predominantly expressed in the brain is enzymatically processed via two routes. Thus, it can be cleaved either by the α-secretase followed by γ-secretase, resulting in the release of soluble APPα through the non-amyloidogenic pathway or by β-secretase followed by γ-secretase, leading to the formation of the highly insoluble Aβ_1-42_ (composed of 42 amino acids) peptide through the amyloidogenic pathway [34,37] (Figure 1). Among the various Aβ isoforms found in the CSF, the levels of Aβ_40_ and Aβ_42_ are the most reliable in terms of assessment for AD diagnosis. Specifically, as Aβ_42_ aggregates into fibrils and plaques within the brain, its concentration in the CSF is considerably reduced, thus serving as an AD indicator [43,44]. However, while Aβ_40_ is the most abundant isoform, there are no significant changes in its levels in AD patients. In this case, its levels are analyzed by the Aβ_42_/Aβ_40_ ratio, which is more reliable than only assessing Aβ_42_ concentrations due to individual fluctuation compensations [35,42,45]. Specifically, the Aβ_42_ values are normalized, as Aβ_40_ is used as a proxy for the total Aβ values [46]. Moreover, other truncated forms of the Aβ_42_ amyloidogenic peptide, including Aβ_37_, Aβ_38_ and Aβ_39_, could provide additional diagnostic information. Among them, the accuracy of the Aβ_42_/Aβ_38_ ratio is comparable to that of Aβ_42_/Aβ_40_ in terms of predicting AD [35,46]. Evidence for these findings relies on autopsy studies, antemortem lumbar CSF analyses, and functional imaging studies based on positron emission tomography using Aβ ligands, e.g., 11c-labelled Pittsburgh Compound [42]. Nonetheless, the use of these biomarkers in routine clinical practice is still in its infancy [40], as there are still some limitations that must be overcome, such as the interindividual differences in the production of Aβ or the overlapping between CSF and Aβ_1-42_ between neurodegenerative disorders, as in Creutzfeldt–Jakob disease, dementia with Lewy bodies, frontotemporal lobar degeneration, and prodromal and manifest (subcortical) vascular dementia [47,48]. As such, most studies analyze Aβ levels in comparison with T-tau and P-tau values in order to increase accuracy [35]. Furthermore, several studies have investigated the levels of APP cleavage metabolites, including soluble APPα, soluble APPβ and total soluble APP, as biomarkers for AD. However, the results are generally inconsistent due to several reasons, including disease heterogeneity, co-morbidities, assay specificity and sensitivity, antibody cross-reactivity, sampling differences, and CSF processing and storage [49,50]. In this context, one study suggested higher levels of soluble APPα in MCI-AD patients compared with non-AD and control groups, and higher levels of APPβ in both AD and MCI-AD patients [50]. Moreover, soluble APPα and soluble APPβ levels have been associated with biomarkers of BACE1 activity.

**Figure 2:**
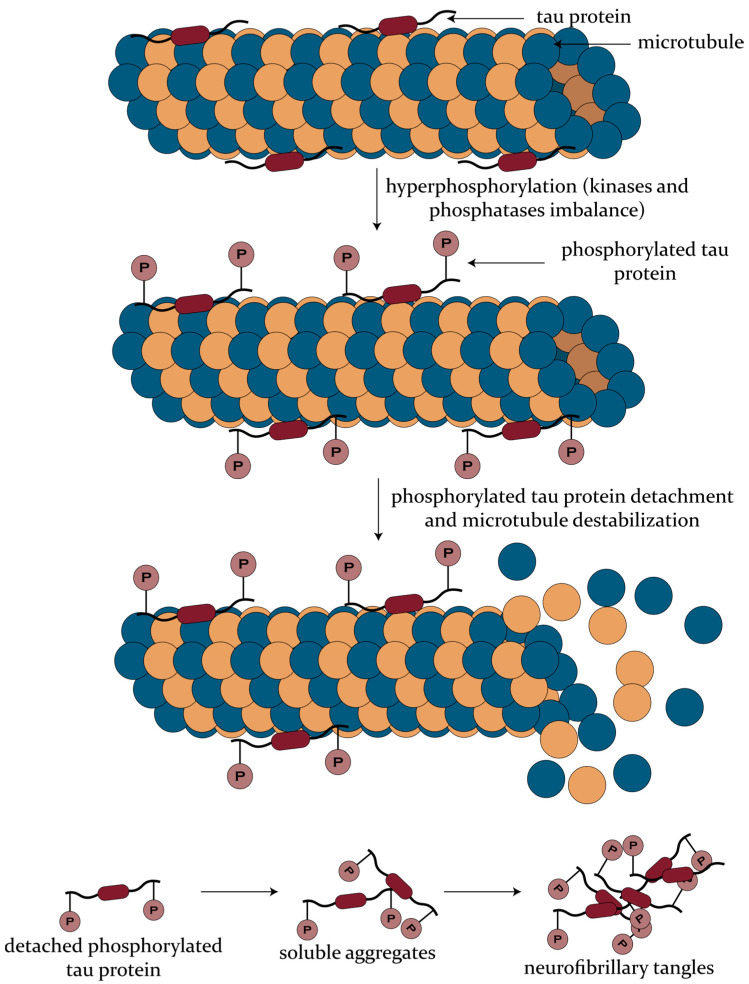
The formation of neurofibrillary tangles through the process of tau protein hyperphosphorylation.

Tau is a highly important microtubule-regulating protein abundantly expressed in the cytosol of axons. Its activity mainly focuses on microtubule-related functions, namely tubulin assembly promotion, dynamic instability regulation, a spatial organization in a parallel network, and axonal transport of kinesins and dyneins, which contribute to microtubule stabilization [37,51,52]. The kinase and phosphatase imbalances in AD lead to the hyperphosphorylation of tau and its consequent detachment from microtubules and accumulation into NFTs (Figure 2). Subsequently, tau and P-tau proteins are released into the extracellular space of the CSF, resulting in increased levels characteristic for neurodegeneration [37]. On one hand, tau proteins are assessed by using monoclonal antibodies, which detect all isoforms independently of their phosphorylation state. In AD patients, T-tau concentrations increase by 200–300%, which is further associated with the severity of neuronal/axonal damage and neurodegeneration. However, increased levels of T-tau have also been observed in other neurological disorders, including stroke, brain trauma, or Creutzfeldt–Jakob disease [53,54,55], which makes it less specific for AD. On the other hand, moderately increased levels of P-tau proteins are more accurately associated with AD, as they indicate both the brain phosphorylation state and the NFTs’ formation and load [35,42].

While the deposition of Aβ plaques occurs years or even decades before the onset of the symptoms, and could be used for early diagnosis, tau biomarkers change later as the disease progresses, and are strongly correlated with local degeneration and cognitive decline [37,56]. The most effective strategy for developing a biomarker-based diagnostic tool is to combine both disease-specific and non-specific biomarkers. In this context, the decrease in Aβ_42_, and concomitant increase in Aβ_42_/Aβ_40_ and Aβ_42_/Aβ_38_ ratios and T-tau and P-tau levels is commonly referred to as the Alzheimer profile or signature, as it offers the possibility of detecting AD in its early stages [35,42,57,58]. Additionally, their combined use for AD diagnosis is characterized by sensitivity and specificity of approximately 85–95% [59]. Similarly, by increasing the palette of biomarkers, the discrimination between AD and other differential diagnoses, such as MCI, dementia or depression, could be possible.

In this context, recent years have witnessed the rise of a new generation of biomarkers related to AD pathological mechanisms, such as neurofilament light (NFL) for neuronal injury, neurogranin, BACE1, SNAP-25 and synaptotagmin for synaptic dysfunction and/or loss, and sTREM2 and YKL-40 for neuroinflammation, due to the activation of microglia and astrocytes [38,60,61].

The neurofilament heteropolymers are the primary cytoskeleton proteins predominantly found in axons. Among the four subunits, namely the three isoforms NFL, neurofilament medium and neurofilament heavy, and alpha-internexin, NFL is the most abundant [59,62]. Forming the core of the neurofilament, NFL is a triplet protein essential to the structure of the myelin that surrounds the axons within the central nervous system [62,63]. As their presence within the CSF is specific for axonal injury, elevated NFL concentrations have been widely reported in neurodegenerative disorders, especially in AD patients [59,62,63,64]. While the mechanisms of NFL aggregation are still unelucidated, they are thought to be similar to the hyperphosphorylation process of tau proteins [65].

Neurogranin is a small neuron-specific and post-synaptic protein abundantly expressed in the brain, especially in the hippocampal and cerebrocortical dendritic spine [66,67,68]. Neurogranin has been found to play key roles in synaptic plasticity and long-term potentiation as a major regulator of the calcium-binding protein calmodulin and of calcium-signal transduction and memory formation [66,67,68,69]. Autopsy studies revealed a possible correlation between neurogranin and AD, as analyses showed reduced levels of neurogranin in brains and increased levels in the CSF of AD patients [68]. In this regard, there is accumulated evidence confirming the potential of neurogranin as an AD biomarker, both as a full-length molecule and as fragments from the C-terminal half [66,70]. Moreover, it has been shown to be able to detect early-stage pathological changes, even in the MCI stage, and predict and monitor AD-related cognitive decline, thus serving as a promising pre-symptomatic biomarker [67,68]. BACE1 (β-site APP cleaving enzyme-1) is an aspartyl protease discovered in 1999, which, by contrast to other peptidases of the pepsin family, such as cathepsin D and E, is a type I transmembrane protein [69,71]. Commonly expressed in neurons, oligodendrocytes and astrocytes, BACE1 is more abundantly found within certain neuronal cell types [69]. The generation of Aβ monomeric forms is dependent upon the activity of BACE1, this being directly related to synaptic functions, plasticity and homeostasis [69,72]. Studies have shown the significantly increased concentrations and activity rates of BACE1 in AD brains and CSF, which is thought to cause a vicious cycle by producing Aβ peptides near synapses [69,72,73,74,75]. Other synaptic dysfunction-associated biomarkers for AD include synaptotagmin, a calcium sensor protein, SNAP-25, a component of the soluble N-ethylmaleimide sensitive factor attachment protein receptor complex, GAP-43, a pre-synaptic membrane protein, and synaptophysin, which has exhibited increased levels in the CSF of AD patients [69,76].

On one hand, TREM2, the triggering receptor expressed on myeloid cells 2, is a type I transmembrane receptor protein of the innate immune system, selectively expressed on the plasma membrane of microglia and dendrocytes within the central nervous system [77,78,79,80,81]. TREM2 plays fundamental roles in microglial functions, including in the phagocytosis of apoptotic neurons, damaged myelin and amyloid plaques, biosynthetic metabolism, proliferation, migration, survival, cytokine release, lipid sensing, and inflammatory signaling inhibition, and it has been proven to be essential in synapse pruning during early development [79,80,81]. Furthermore, its ectodomain is cleaved in the cell surface and shed at the plasma membrane, thus releasing a soluble fragment (sTREM2) which can be measured in the CSF as an indicator of microglial activity [78,79,80]. As it is involved in the regulation of microglia dynamics, and the subsequent amyloid plaque formation and synaptic plasticity, increased levels of sTREM2 within the CSF have been related to a protective response against AD pathology, thus serving as a potential biomarker [80,81,82,83]. On the other hand, YKL-40, the inflammation-related glycoprotein known as chitinase-3-like protein 1, breast regression protein 39, human cartilage glycoprotein 39 or chondrex, belongs to the family of chitinase-like proteins, but lacks the enzymatic activity of chitinases [84,85]. Normally expressed in the fibrillar astrocytes within the white matter, YKL-40 plays key roles in inflammation, proliferation, angiogenesis and tissue remodeling [84,85,86], and CSF YKL-40 is a biomarker for astroglial activity [80]. Furthermore, elevated levels of YKL-40 in the brain and CSF are generally associated with neurodegeneration, appearing as a pre-clinical sign of AD pathology [80,86,87].

Another important group of AD biomarkers is the microRNAs (miRNAs), which are small non-coding RNAs with an average length of 22 nucleotides, involved in gene expression at the post-transcriptional level, regulation through binding to mRNA targets, and the subsequent translational repression or degradation of the target by the RNA-induced silencing complex [88,89,90]. Although recent studies have been intensively focusing on miRNA deregulation associated with AD, the lack of standardization in the quantification methods and protocols used is considerably challenging for establishing the discrimination power of miRNAs as biomarkers for AD [91]. Thus, the available results are generally not comparable since they target different miRNA molecules, which further increases the complexity of the subject [91,92,93,94,95,96].

The previously described CSF biomarkers for AD and the associated mechanisms of pathology are summarized in Table 1.

## 3. Blood Biomarkers

Although CSF biomarkers provide significantly more accurate diagnostics of AD and/or MCI, their clinical application is generally limited due to their invasive nature, which is traumatic to patients, and their high costs. Therefore, the scientific focus has shifted towards more accessible biomarkers that could increase their application for clinical practice. In this context, the use of peripheral blood biomarkers for AD diagnosis possesses a series of advantages, namely minimal invasiveness, facile sampling, cost- and time-efficiency, and widespread adoption [67,105].

Nonetheless, although blood communicates with the brain through the blood–brain barrier, the lymph vessels and the glymphatic system, the interchange is indirect. Therefore, the applicability of blood biomarkers in clinical practice is still not possible due to a series of challenges, in terms of both biological and technical issues [60,105,106,107]. First, the central nervous system is an isolated environment and the concentration of the potential biomarkers might be relatively low, as they must cross the blood–brain barrier as intact molecules [106]. Additionally, the volume ratio between the blood and the CSF will cause a significant analyte dilution [60]. However, there is strong evidence of barrier dysfunction in AD patients, which leads to increased protein and other molecule exchanges [105,106]. Second, as blood is a highly complex fluid comprising various molecules and cells, non-specific biomarkers, such as inflammatory or acute phase proteins, could be expressed by sources other than the central nervous system, which further introduces and increases variability within analyses [60,106]. Additionally, the variety of proteins and heterophilic antibodies present in the blood might potentially cause interference in the analysis [60]. Third, blood biomarkers might undergo liver or plasma proteolytic degradation, matrix effects due to plasma protein or blood cell adhesion, or kidney excretion, which will further substantially lower their concentration [60,106]. Fourth, the sensitivity and specificity of blood biomarkers are still considerably low, as there is a high risk of the overlapping of neurodegenerative disorders and other co-morbidities of AD patients that could also change plasma protein profiles [67,106]. Therefore, blood biomarker assays for AD diagnosis still lack standardization between instruments and laboratories, and the complexity of the blood is associated with a series of variables that are challenging in terms of result replication [60,105].

Among the conventional biomarkers for AD, Aβ_42_, Aβ_40_ and Aβ_42_/Aβ_40_ have been recognized as potential screening molecules [108]. However, early studies led to inconsistency between results and a lack of correlation between CSF and blood Aβ [46,106,109,110,111]. Such results were probably due to low Aβ concentrations in blood and the influences of matrix effects, as plasma proteins have a tendency of binding to Aβ, and the analytical sensitivity of the assay did not allow for diluting these effects [46,109], as measurements were performed using enzyme-linked immunosorbent assay (ELISA) methods [106,110,112]. However, more recent studies are considerably more promising, as they use ultrasensitive immunoassay techniques, such as single-molecule array or SIMOA, immunoprecipitation coupled with mass spectrometry, and stable isotope labeling kinetics followed by immunoprecipitation coupled with mass spectrometry [46,105,106,109,110,111]. As such, the results showed the expected decrease in blood Aβ_42_, Aβ_40_ and Aβ_42_/Aβ_40_ levels in AD and MCI patients [106,109,110,111,113]. However, the presence of various factors that introduce variability to the results limits the applicability of Aβ as a blood biomarker for AD [105].

Similarly, the introduction of ultrasensitive immunoassay techniques, including SIMOA, mesoscale discovery or MSD, label-free real-time surface plasmon resonance technology, and immunomagnetic reduction, has led to more promising results in terms of T-tau and P-tau blood levels. In this regard, results have shown that increased levels of blood T-tau and P-tau are generally associated with AD [46,106,109]. However, more accurate results have been obtained by using enzymes involved in tau protein hyperphosphorylation processes, such as glycogen synthase kinase 3β (GSK-3β) and dual-specificity tyrosine-phosphorylation regulated kinase A (DYRK1A) [105]. On one hand, GSK-3β is a GSK-3 isoform, part of the serine/threonine kinase family, known for its important roles in neuron polarity and synapse plasticity. Consequently, there is strong evidence of its implications in the pathological mechanisms of neurodegeneration disease development and the progression of tauopathies associated with AD [114,115,116,117]. In this context, blood levels of GSK-3β are considerably elevated in AD and MCI patients, which proves its potential as a blood-based AD biomarker [105,118]. On the other hand, DYRK1A, a member of the proline-directed serine/threonine kinases, is widely known for its implications for cell proliferation, as well as various signaling pathways fundamental for brain development and function, namely neuron survival, synaptic plasticity, and actin cytoskeleton and microtubule regulation [119,120,121]. As AD patients present considerably reduced blood levels, DYRK1A could be used as a potential biomarker [105,122].

The emergence of the ultrasensitive techniques has also allowed for the accurate quantification of blood NFL, which has been shown to closely correlate with CSF results, thus reflecting brain pathology [46,106,110]. Both plasma and serum levels of NFL are elevated in AD and MCI patients years before symptom onset [46,105,109,110,111,123,124]. Additionally, as NFL levels could also serve as biomarkers for disease severity, namely brain atrophy, cognitive impairment or glucose hypometabolism, it can also be used as a biomarker for disease staging [46]. Although it is among the most consistent blood biomarkers [111,123,125], increased concentrations of NFL are not specific for AD, as they have been observed in other neurodegenerative disorders [105,109].

Moreover, several studies have demonstrated that sustained chronic inflammation is directly related to AD development, as postmortem tissues of AD models exhibited inflammatory responses [126]. Among the mediators involved in the systemic immune response regulation, including transcriptional factors, cytokines, chemokines, complements, coagulation factors, enzymes, various peptides and lipids [127], interleukins (IL-1, IL-4, IL-6 and IL-10), cytokine I-309, interferon-γ, and tumor necrosis factor α (TNF-α) are particularly important biomarkers for the early diagnosis of AD [105].

Furthermore, clusterin, also termed as apolipoprotein J, is a highly sialylated multifunctional glycoprotein that is highly expressed in the brain, liver, testicles and ovaries [128,129,130]. Studies show that clusterin is involved in a series of pathophysiological states, including cell death, oxidative stress, proteotoxic stress and neurodegenerative processes [130]. As its main function is to act as a chaperone for various extracellular proteins, it has been demonstrated that clusterin is capable of binding Aβ peptides, thus decreasing Aβ toxicity and the associated apoptosis and oxidative stress [105,130,131]. In this context, as it is found in higher concentrations in the blood of AD patients, clusterin could be a promising AD biomarker [105].

The previously described blood biomarkers for AD and the associated mechanisms of pathology are summarized in Table 2.

## 4. Saliva Biomarkers

Saliva is a complex biological fluid secreted in the mouth by three main pairs of salivary glands, namely the parotid, the submandibular, and the sublingual, which generate 0.75–1.5 L daily. The compositions of their secretions depend on the sympathetic and parasympathetic stimulation, circadian rhythm, health status, eating habits and drug intake [138,139]. Considering the direct relation between the salivary gland and the nervous system, as the facial nerve innervates the sublingual and submandibular glands through the submandibular ganglion and the glossopharyngeal nerve innervates the parotid gland through the otic ganglion, saliva could represent an important source of biomarkers for nervous system disorders [139,140]. In contrast to blood, saliva is a matrix that can be collected easily and non-invasively, at all ages and many times per day, and assessed through different assays [139,141,142,143,144], which is promising for its future clinical application in the timely detection, diagnosis, prognosis and monitoring of neurological disorders [142,145]. In this regard, a novel term has been introduced, salivaomics, which encompasses all biomarkers discovered within the genome, microbiome, epigenome, transcriptome, proteome and metabolome for the development of translational and clinical tools for diagnosis [145,146].

Therefore, due to the capacity of molecules to pass from the blood to the saliva through passive diffusion, active transport or microfiltration, saliva is a promising AD-related biomarker pool that could be used for its early and accurate diagnosis [147,148]. The most important AD biomarkers found within the saliva are Aβ peptides, T-tau and P-tau, acetylcholine, lactoferrin, and trehalose, each related to different AD pathophysiological mechanism.

Owing to the saliva–blood interactions and the buccal cell degradation, Aβ peptides should also be present in the saliva, as APP is a widely expressed protein in the peripheral tissues. Although the number of studies on the matter is still considerably limited, recent results have shown that salivary Aβ_42_ is increased in AD patients, while Aβ_40_ does not change [148,149,150]. However, there are no studies regarding the Aβ_42_/Aβ_40_ ratio in the saliva, which should also be validated considering its significant relevance in the CSF [151].

Similarly, studies on salivary T-tau and P-tau are still limited, with preliminary results demonstrating elevated levels of T-tau and P-tau, and also an elevated P-tau/T-tau ratio [152,153]. However, the results are not conclusive, as tau proteins are also expressed and secreted by acinar epithelial cells, the subunits of salivary glands, and released from the cranial nerves [151,154].

Furthermore, as salivary glands are under cholinergic innervation, acetylcholinesterase, a type-B carboxylesterase enzyme mainly found in the synaptic cleft at the post-synaptic neuromuscular junctions, further diffuses into the saliva [151,155,156]. Its primary function is the termination of neuron transmission and signaling, but recent studies have demonstrated its role in the development of AD by promoting Aβ fibril formation [156,157,158]. In this context, the available studies reported reduced levels of salivary acetylcholinesterase associated with aging and even lower levels for AD patients [139,148,151]. However, while they proved its potential as a salivary AD biomarker, the conclusiveness of the results is still limited due to a lack of standardization [151].

Antimicrobial peptides have been previously proposed as biomarkers for brain infections involved in the AD developmental processes [151]. An example of such biomarkers is lactoferrin, a globular non-hemic iron-binding glycoprotein that belongs to the family of serum transferrin proteins, and it is mostly synthesized by glandular epithelial cells and neutrophils [159,160,161,162]. Owing to its iron-binding activity, lactoferrin is a multifunctional protein that exhibits antibacterial, antiviral, antifungal, antioxidant, immunomodulatory, anti-cancer, anti-inflammatory and anti-allergenic properties [160,162,163,164]. Moreover, while there is evidence of lactoferrin presence within the human brain, its levels are substantially increased in AD patients and those with related neurodegenerative disorders, which could be attributed to its Aβ-binding ability [165,166,167]. Therefore, lactoferrin has been associated with AD pathogenesis, as it has been detected in the amyloid plaques, NFTs and microglia of AD brains [164,165]. Studies on AD patients are still limited, but there is strong evidence that the salivary levels of lactoferrin significantly decrease when compared to healthy controls and elderly subjects [139,148,164,168]. Moreover, lactoferrin has also demonstrated its potential for early disease detection, as the accuracy of AD diagnosis using it was greater than with CSF T-tau and Aβ42 [139,151].

The previously described saliva biomarkers for AD and the associated mechanisms of pathology are summarized in Table 3.

## 5. Emerging Body Fluid Biomarkers

Recent years have witnessed significant advancements in the profiling technologies, which have improved the detection sensitivity and allowed for the quantification of minute samples. In this manner, previously difficult-to-assess body fluids, such as urine, tears or olfactory fluids, have become a rich source of biocompounds that could reflect the pathological state of an individual [171].

Urine has become a highly desirable source of disease biomarkers, as it can easily and non-invasively be collected in relatively large volumes. Additionally, it contains cellular components, biochemical compounds, and proteins originating from plasma glomerular filtration, renal tubule excretion or urogenital tract secretion, thus reflecting the metabolic and pathophysiological condition of an individual. In this context, recent works have focused on the plethora of biomarkers present within the urinary proteins, glycoproteins and exosomes that could allow for the early diagnosis, prognosis, prevention or treatment of various diseases [172,173]. Furthermore, urine can also reflect AD pathology signs, generally associated with modifications in protein and lipid metabolism caused by oxidative stress [174,175]. Moreover, since the concentration of the creatinine waste product is physiologically stable, it can be used for normalizing urine biomarker concentrations [174]. In this context, the most promising urinary biomarkers include isoprostane [176], glycine and total free amino acids [177], and 8-hydroxy-2‘-deoxyguanosine [178], which have achieved over 90% accuracy [174,175].

While tears are available in considerably reduced volumes for sampling, they are a neglected key reservoir of biomarkers, with great potential in medical diagnostics [179,180]. Tears are complex protein, lipid, mucin, water and salt mixtures, and the development of novel proteomic, lipidomic and glycomic techniques has allowed for a complete understanding of these components and their changes associated with ocular or non-ocular disorders [171,181,182]. For example, proteomic techniques have revealed the presence of AD-related peptides within aqueous humor samples [183], while tear fluid has proven to be clinically relevant through the discovery of a combination of four tear proteins, namely lipocalin-1, dermicidin, lysozyme C and lactritin, with a sensitivity of 81% and a specificity of 77% for AD [184,185]. Another study suggested the discriminatory power of tear T-tau and Aβ_42_, as their levels increased in AD patients [186]. Additionally, total microRNA abundance was also found at increased levels in AD patients, with microRNA-200b-5p as the most promising AD biomarker [183,187].

Moreover, several studies have reported the isolation of NFTs and identified increased levels of T-tau and P-tau in AD patients’ nasal secretions [35], thus proving the potential of olfactory fluids as non-invasive AD biomarkers.

## 6. Conclusions and Future Perspectives

AD is the most common neurodegenerative disorder that predominantly affects the elderly population. Thus, it is expected that the number of AD patients worldwide will reach 60 million by 2030, which will have a significant impact on the global health system. The current diagnosis methods involve cognitive tests, neuroimaging techniques and CSF assays. However, there is still no clinical strategy available for the accurate and early detection of AD. Recent trends have focused on identifying novel biomarkers beyond Aβ and tau proteins, as well as new matrices as biomarker sources, such as the blood, saliva, urine, tear or olfactory fluids. While there have been considerable advancements in the field, the lack of standardized sampling and assays poses significant challenges for the use of such biomarkers in the clinical practice. In this context, recent trends have been focusing on the identification of protein or lipid panels, which could better reflect the complete mechanisms of AD.

Furthermore, research should also focus on the development of advanced platforms and biosensor devices that could provide real-time information regarding the health status of AD patients [188]. In this context, biosensor-on-chip devices could represent a promising strategy for accurately assessing a great pallet of AD biomarkers.

## Figures and Tables

**Table 1:** The major changes of the identified CSF biomarkers for AD and the associated mechanisms of pathology.

Mechanism of AD Pathology	CSF Biomarker	Change in AD Pathology	Sensitivity	Specificity	References
Aβ plaque deposition	Aβ_42_	↓	0.69–0.81	0.44–0.89	[43,97,98,99,100]
	Aβ_40_	-	0.72	0.39	[98,101]
	Aβ_38_	-	0.63	0.56	[98,101]
	Aβ_42_/Aβ_40_	↑	0.81–0.93	0.60–1	[45,97,98,99]
	Aβ_42_/Aβ_38_	↑	0.92	0.89	[101]
tau pathology	T-tau	↑↑	0.74–0.77	0.70–0.75	[97,100]
	P-tau	↑	0.66–0.73	0.63–0.82	[97,100]
neuronal injury	NFL	↑	0.81	0.79	[100]
synaptic dysfunction and/or loss	neurogranin	↑	0.73	0.84	[102]
	BACE1	↑	0.87	0.63	[103]
	synaptotagmin	↑	n.r.	n.r.	[69,76]
	SNAP-25	↑	n.r.	n.r.	[69,76]
	GAP-43	↑	n.r.	n.r.	[69,76]
	synaptophysin	↑	n.r.	n.r.	[69,76]
neuroinflammation	sTREM2	↑	n.r.	n.r.	[82,83]
	YKL-40	↑	0.77–0.85	0.81–0.84	[80,86,87,104]

n.r.—not reported; ↓—decrease; ↑—increase; ↑↑—high increase.

**Table 2:** The major changes of the identified blood biomarkers for AD and the associated mechanisms of pathology.

Mechanism of AD Pathology	Blood Biomarker	Change in AD Pathology	Sensitivity	Specificity	References
Aβ plaque deposition	Aβ_42_	↓	0.82	0.77	[35,132,133]
	Aβ_40_	↓	n.r.	n.r.	[133]
	Aβ_42_/Aβ_40_	↓	0.75	0.77	[35,132,133]
tau pathology	T-tau	↑	0.62	0.54	[134]
	P-tau	↑	n.r.	n.r.	[135]
	GSK-3β	↑	n.r.	n.r.	[105,118]
	DYRK1A	↓	n.r.	n.r.	[105,122]
neuronal injury	NFL	↑	0.86	0.76	[136]
inflammation	IL-1, IL-4, IL-6, and IL-10	↑	n.r.	n.r.	[105]
	cytokine I-309	↑	n.r.	n.r.	[105]
	interferon-γ	↑	n.r.	n.r.	[105]
	TNF-α	↑	n.r.	n.r.	[105]
apoptosis	clusterin	↑	0.76	0.63	[105,137]

n.r.—not reported; ↓—decrease; ↑—increase.

**Table 3:** The major changes of the identified saliva biomarkers for AD and the associated mechanisms of pathology.

Mechanism of AD Pathology	Saliva Biomarker	Change in AD Pathology	Sensitivity	Specificity	References
Aβ plaque deposition	Aβ_42_	↑	0.16	0.93	[148,149,150,169]
	Aβ_40_	-	n.r.	n.r.	[148,149]
	acetylcholinesterase	↓	n.r.	n.r.	[139,148,151,170]
tau pathology	T-tau	↑	n.r.	n.r.	[152,153,171]
	P-tau	↑	n.r.	n.r.	[152,153]
	P-tau/T-tau	↑	0.73–0.83	0.30–0.50	[152,153]
inflammation	lactoferrin	↓	1	0.98	[168]

n.r.—not reported; ↓—decrease; ↑—increase.
